# Looking for Image Statistics: Active Vision With Avatars in a Naturalistic Virtual Environment

**DOI:** 10.3389/fpsyg.2021.641471

**Published:** 2021-02-22

**Authors:** Dominik Straub, Constantin A. Rothkopf

**Affiliations:** ^1^Institute of Psychology, Technical University of Darmstadt, Darmstadt, Germany; ^2^Centre for Cognitive Science, Technical University of Darmstadt, Darmstadt, Germany

**Keywords:** visual perception, efficient coding, natural image statistics, virtual environments, virtual agents, active vision

## Abstract

The efficient coding hypothesis posits that sensory systems are tuned to the regularities of their natural input. The statistics of natural image databases have been the topic of many studies, which have revealed biases in the distribution of orientations that are related to neural representations as well as behavior in psychophysical tasks. However, commonly used natural image databases contain images taken with a camera with a planar image sensor and limited field of view. Thus, these images do not incorporate the physical properties of the visual system and its active use reflecting body and eye movements. Here, we investigate quantitatively, whether the active use of the visual system influences image statistics across the visual field by simulating visual behaviors in an avatar in a naturalistic virtual environment. Images with a field of view of 120° were generated during exploration of a virtual forest environment both for a human and cat avatar. The physical properties of the visual system were taken into account by projecting the images onto idealized retinas according to models of the eyes' geometrical optics. Crucially, different active gaze behaviors were simulated to obtain image ensembles that allow investigating the consequences of active visual behaviors on the statistics of the input to the visual system. In the central visual field, the statistics of the virtual images matched photographic images regarding their power spectra and a bias in edge orientations toward cardinal directions. At larger eccentricities, the cardinal bias was superimposed with a gradually increasing radial bias. The strength of this effect depends on the active visual behavior and the physical properties of the eye. There were also significant differences between the upper and lower visual field, which became stronger depending on how the environment was actively sampled. Taken together, the results show that quantitatively relating natural image statistics to neural representations and psychophysical behavior requires not only to take the structure of the environment into account, but also the physical properties of the visual system, and its active use in behavior.

## 1. Introduction

One of the most successful and long-standing computational approaches to perception has posited that perceptual systems are adapted to the sensory stimuli they encounter. This idea was originally fueled by the advent of information theory (Shannon, 1948) and dates back to the 1950s, when Attneave (1954) and later Barlow (1961) argued that sensory systems should represent their input in a way that removes statistical redundancy while retaining as much information as possible. Due to the structure in the environment to which the sensory system is exposed, not all conceivable inputs are equally likely. Since the computational resources available to a sensory system are limited by biological constraints, more resources should be allocated to process the inputs that are more likely to be encountered. This formalization is closely related to Bayesian approaches to perception (Knill and Richards, 1996), according to which the visual system infers the most likely causes of ambiguous, uncertain, and noisy sensory signals it obtains by probabilistically combining them with prior knowledge. In this Bayesian setting, computing a posterior probability over image causes only leads to the correct inferences, if the prior distribution over these image causes is adapted to the empirical distribution of the variables in the environment. Thus, it is of crucial importance both in the framework of information theory and the Bayesian framework for a sensory system to be well-calibrated to the statistics of its input (Fiser et al., 2010; Ganguli and Simoncelli, 2014; Wei and Stocker, 2017).

A first requirement in quantitatively establishing the link between neural representations and perception on one hand and environmental statistics on the other hand (Simoncelli and Olshausen, 2001) is therefore to analyze the statistics of the natural input to the visual system, i.e., the typical kinds of stimuli it encounters while an organism interacts with its natural environment. But, what are the stimuli that sensory systems encounter? Regarding the visual system, this line of thought has motivated numerous studies investigating environmental statistics via natural image ensembles. Classic studies typically used images from natural scene databases, which mostly consist of photographs taken in natural landscapes. Early studies used the database by van Hateren and van der Schaaf (1998), which is made up of 4,000 monochrome, calibrated images of natural landscapes in part containing human artifacts including roads, buildings, and cars. Several other natural image datasets have been collected over the decades with the specific goal of measuring image feature statistics and relating them to properties of the human visual system. Such datasets include image collections from the putative birthplace of homo sapiens (Tkačik et al., 2011) and images together with depth measurements of natural environments (Adams et al., 2016) or video sequences recorded by placing cameras in the natural habitat of animals (Qiu et al., 2020) or by simulating some physical properties of the visual system of animals (e.g., Nevala and Baden, 2019; Tedore and Nilsson, 2019).

There are, however, several aspects in which photographic images of natural environments and the natural input to the visual system differ. Betsch et al. (2004) identified two of these aspects: First, the temporal dynamics of the environment are lost when considering only static images. Second, images selected by humans potentially introduce biases, since the process by which the visual system samples the environment is most likely different from the selection process for these databases. Humans and animals actively direct their eyes to different parts of the scene in conjunction with the ongoing behavior (Land and Tatler, 2009), which is primarily determined by behavioral goals (Hayhoe and Ballard, 2005). During walking in natural environments humans show specific preferences in gaze directions (Einhäuser et al., 2007; Pelz and Rothkopf, 2007). When navigating rough terrains, subjects tend to align their gaze most of the time with environmental features lying in their plane of progression (Hollands et al., 2002) and they differentially adjust their gaze targets to obstacles and targets along the way (Rothkopf et al., 2007). Because of this active selection of gaze targets, the local statistics at the point of gaze vary between tasks (Rothkopf and Ballard, 2009). This suggests, that the statistics of the natural input to the visual system depend on the active sensory strategy. Indeed, a preliminary study revealed, that preferred orientations of receptive fields learned with sparse coding differ depending on looking directions (Rothkopf et al., 2009). Several studies have approached this by placing cameras on the head of animals and humans moving through natural environments (e.g., Betsch et al., 2004; Schumann et al., 2008; Orhan et al., 2020).

Another important difference was noted by Pamplona et al. (2013). The field of view in natural image databases is typically limited, e.g., 26° in the van Hateren and van der Schaaf (1998) database. Thus, the statistics of natural images are often assumed to be homogeneous across the visual field or even space-invariant, which previous research has shown to not be the case (Bruce and Tsotsos, 2006; Rothkopf et al., 2009; Pamplona et al., 2013). Furthermore, the photoreceptors at the back of the retina are not arranged on a plane but on an approximately spherical surface. By simply using camera images, one assumes that the geometrical optics of the eye are identical to those of a camera or that these differences do not matter. Pamplona et al. (2013) inspected the power spectra of images after taking into account the human eye's geometrical optics and the blurring introduced by the eccentricity-dependent modulation transfer function (Navarro et al., 1993) and found eccentricity-dependent effects of both the geometry and blurring on the distribution of spatial frequencies. Thus, the sensory stimuli that visual systems encounter may not be well represented by photographic images but are influenced by the respective imaging process and the active use of the visual system. The crucial question resulting from this observation is, whether these factors significantly influence the statistics of the sensory signals entering the visual system or whether they do not matter.

Here we investigate quantitatively through simulations of avatars in virtual reality whether differences in the imaging system and in visual exploration strategies do actually influence image statistics across the visual field or not. Specifically, we generate parametric image databases for two visual systems by moving a human and a cat avatar through a virtual wooded environment and selecting gaze targets according to three different visual strategies. Using virtual scenes might seem like a step away from analyzing natural image statistics. However, virtual environments created in game engines have several important advantages over natural image databases. The properties of the environment are controlled, which makes the dataset easily reproducible (Rothkopf et al., 2009; Richter et al., 2016). It also allows full control over the imaging process, including focal length, field of view, and eye height. This allows capturing images with a much larger field of view than well-known image databases (e.g., van Hateren and van der Schaaf, 1998). Furthermore, by controlling the active sampling strategy of gaze targets and the process of image formation on the model retina, we can quantify their influence on image statistics. Finally, previous research has shown that while differences between simulated images and real-world images for certain vision tasks exist (Veeravasarapu et al., 2017), commonly considered image statistics relevant for the analysis of visual systems such as second-order luminance statistics and Gabor filter responses can match those obtained on classic images databases (Rothkopf and Ballard, 2009).

Using virtual environments furthermore allows sampling images from the viewpoint of a human and a cat and project local image patches onto idealized model retinas of the respective species. Recent work comparing the statistics of optic flow in natural videos between infants and their mothers (Raudies et al., 2012; Raudies and Gilmore, 2014) has shown that the viewpoint matters. Here, we compare human and cat viewpoints, because the cat's primary visual cortex is a common animal model in visual neuroscience and is, despite their differences, often compared to the primate visual system (Payne and Peters, 2001). Thus, the present study contributes to the understanding of the source of potential differences in neuronal representations between these two species. Neurophysiological and neuroimaging studies for example revealed cardinal and radial orientation biases in early visual areas both in cats (Leventhal, 1983; Wang et al., 2003) and humans (Furmanski and Engel, 2000; Sasaki et al., 2006), albeit with quantitative differences. We argue that in order to transfer from a model system to humans, it is important to understand how differences in neural representations arise as a function of different natural input. In this paper, we take a step in this direction by comparing low-level statistics of the input of the human and cat visual system.

While sampling locations in the environment during exploration can still introduce biases, it is a step toward incorporating such explorative biases that occur during natural behavior, instead of those that play a role in choosing photographs for a database. Therefore, positions and gaze directions were recorded at regularly spaced temporal intervals along a trajectory during free exploration of the environment by a single subject. Instead of deliberately taking images in the environment, we opted for this method to avoid the above-mentioned biases in selecting images. In addition to the recorded gaze directions, we considered three different visual strategies: an agent looking straight ahead, an agent looking toward the ground, and an agent with random gaze directions. At each of these recorded positions and for each of these active vision strategies, image patches were sampled across the visual field. The geometric optics of the eye were modeled by approximating the spherical surface of the retina locally with tangential planes, in an approach similar to Pamplona et al. (2013). The second- and higher-order statistics of images obtained according to this process were analyzed by computing the power spectra and edge filter responses. This allows establishing and quantifying the influence of the imaging process and the active use of the visual system on the statistics of the input to the visual system.

Using this virtual image dataset, we are equipped to ask the following questions. Do the image statistics in the virtual environment in the central region of the visual field match those of photographic images? Going beyond the central region, which is covered by common natural image databases, do the image statistics change with increasing eccentricity and across different regions of the visual field and if so, how? Does it make a difference whether we view the environment with the eyes of a cat or a human? And finally, do different active vision strategies lead to differences in image statistics? After all, while quantitative differences in second order image statistics across image categories (Torralba and Oliva, 2003) have been reported, much less is known whether and how the factors investigated in the present study affect image statistics. It is conceivable that differences in visual sampling of the environment would average out in large image ensembles. Indeed, our results show that the simulated images match a number of summary statistics at the center of gaze previously reported in the literature using photographic image databases. Specifically, we find the characteristic 1/*f*^2^ spatial frequency behavior (van der Schaaf and van Hateren, 1996) and a cardinal orientation bias (Coppola et al., 1998). Replicating previous results, we also find cardinal and radial biases in the orientation distributions across the visual field. But, varying the parameters of the imaging process as well as the viewpoint between a human and a cat avatar results in mensurable influences on image statistics. Similarly, image ensembles obtained with different active vision strategies also show clear differences in image statistics. Taken together, these results provide clear evidence, that studying neuronal representations and perceptual properties of the visual system not only needs to quantify statistical properties of natural photographic images but also the statistics imposed on the stimulus by the imaging process and the statistics of active use of the visual system. The dataset on which this study is based will be released as part of this publication to facilitate further investigations of these influences.

## 2. Methods

### 2.1. Virtual Scene Dataset

In order to analyze the properties of the natural input of the visual system, we created a dataset of image patches in a naturalistic virtual environment. Image patches were sampled across a large visual field (120°) from two different viewpoints and with three different active vision strategies. The imaging process was adapted to account for the projective geometry of an idealized retina.

#### 2.1.1. Virtual Environment

The virtual scenes were constructed in Unity^1^, a 3D game engine and integrated development environment. A naturalistic forest environment was created with close to photo-realistic models of trees, undergrowth and smaller plants, deadwood, and rocks based on an interactive Unity demo^2^.

#### 2.1.2. Simulating Virtual Agents' Movement

In this study, we consider static images so that we need to select static positions and viewing directions to obtain individual images instead of movie sequences with naturalistic movement trajectories. Therefore, the first author obtained movement sequences by exploring the virtual environment with no particular goal in mind. The environment was presented in first-person view on a 27” computer screen. While navigating the environment using mouse and keyboard controls, the position and orientation of the viewpoint were recorded every 2 s. In total, 663 positions were recorded.

#### 2.1.3. Simulating Human and Cat Avatars

**Figure 1:**
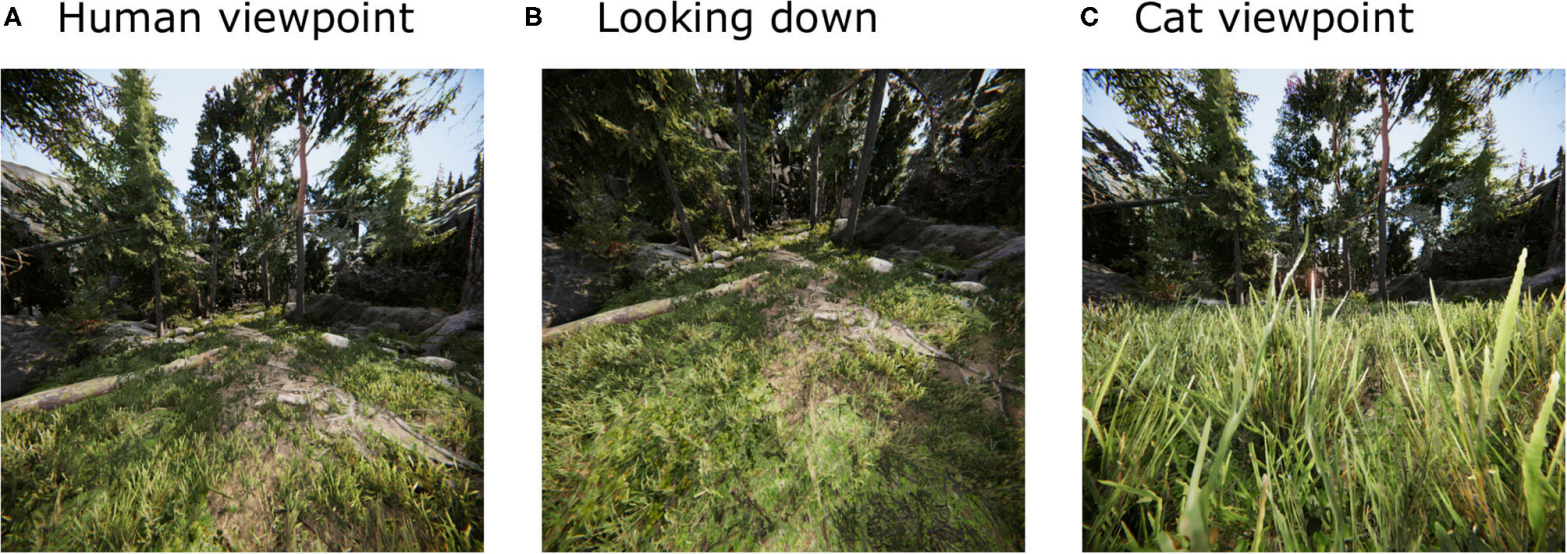
Illustrations of the virtual environment. The virtual environment, shown from the viewpoint of a human looking straight ahead **(A)** and down **(B)** and from the viewpoint of a cat **(C)**. Note that the distortions present in these images result from the projection of a large field of view on a single plane and are different from the geometrical distortions considered in the following (see Figure 4).

As a wealth of classic data on the visual system have been recorded in different animal species (particularly humans, monkeys, and cats), we simulated a human and a cat avatar. This was achieved by selecting a viewing height of 1.8 m for the human avatar and a viewing height of 0.25 m for the cat avatar. The positions (except eye height) and viewing directions were those recorded during exploration of the environment. Furthermore, the parameters of the virtual eye model (see section 2.1.6) were chosen to be realistic for each species. Exemplary screenshots from the two viewpoint heights are shown in Figures 1A,C, respectively.

#### 2.1.4. Simulating Active Vision Strategies

**Figure 2:**
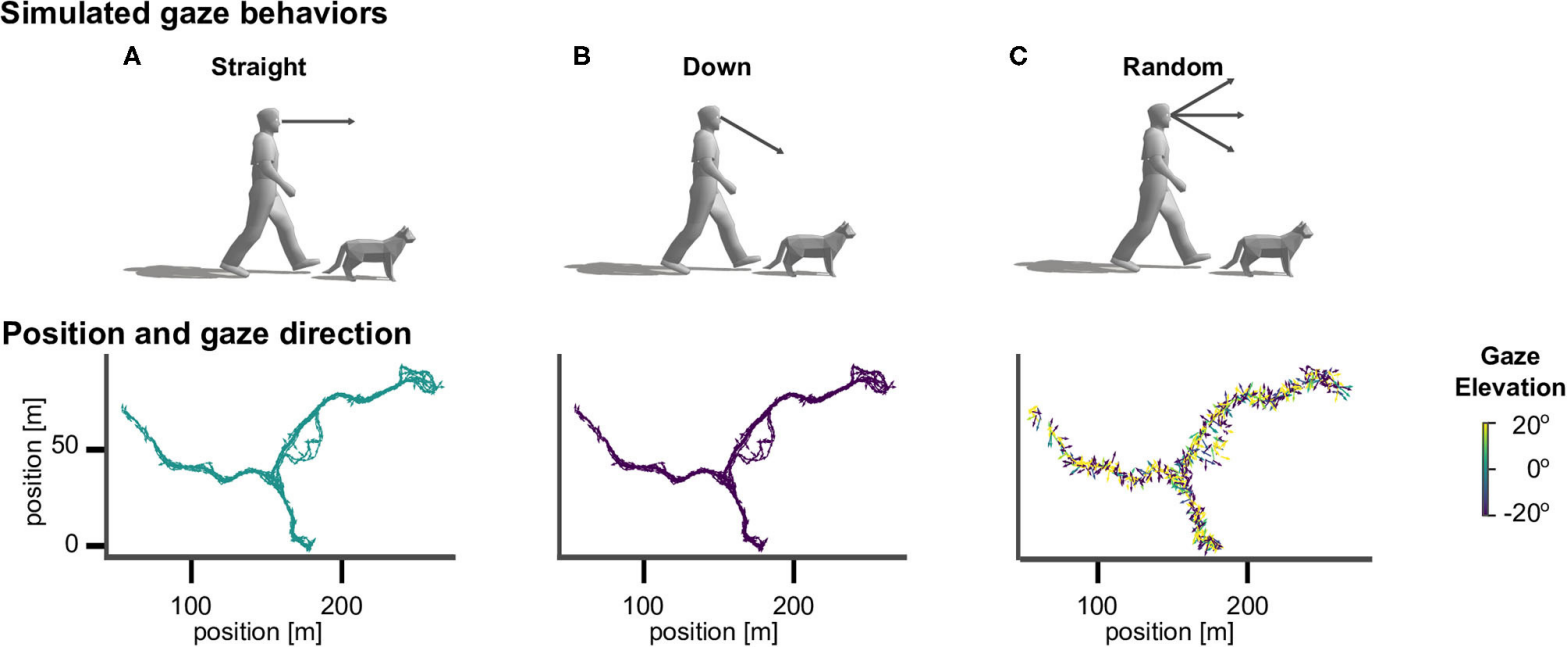
Virtual agents and active gaze behaviors. Top row: illustration of gaze directions in human and cat avatars. **(A)** The “straight” agents were simulated with gaze being directed in the direction of locomotion. **(B)** The “down” agents directed gaze downward from the horizontal heading direction by an angle of 30° . **(C)** The “random” agents directed gaze in random heading and elevation angles. Bottom row: Top view of the corresponding walking trajectory in virtual space and the viewing directions, which are indicated by the direction (heading) and color (elevation) of arrows.

To investigate the effect of different active vision strategies we sampled gaze directions according to three different methods (see Figure 2). The *straight* agent always looked in the heading direction of the movement, with an elevation angle of zero. The *down* agent directed gaze at an elevation angle of 30° downward from the horizontal heading direction, so that the agent always looked to the ground (Figure 1B). The *random* agent directed gaze in random heading and elevation angles, which were drawn from a standard normal distribution (in radian units) centered around the heading direction, which corresponds to a standard deviation of 57.30°.

#### 2.1.5. Imaging Process

**Figure 3:**
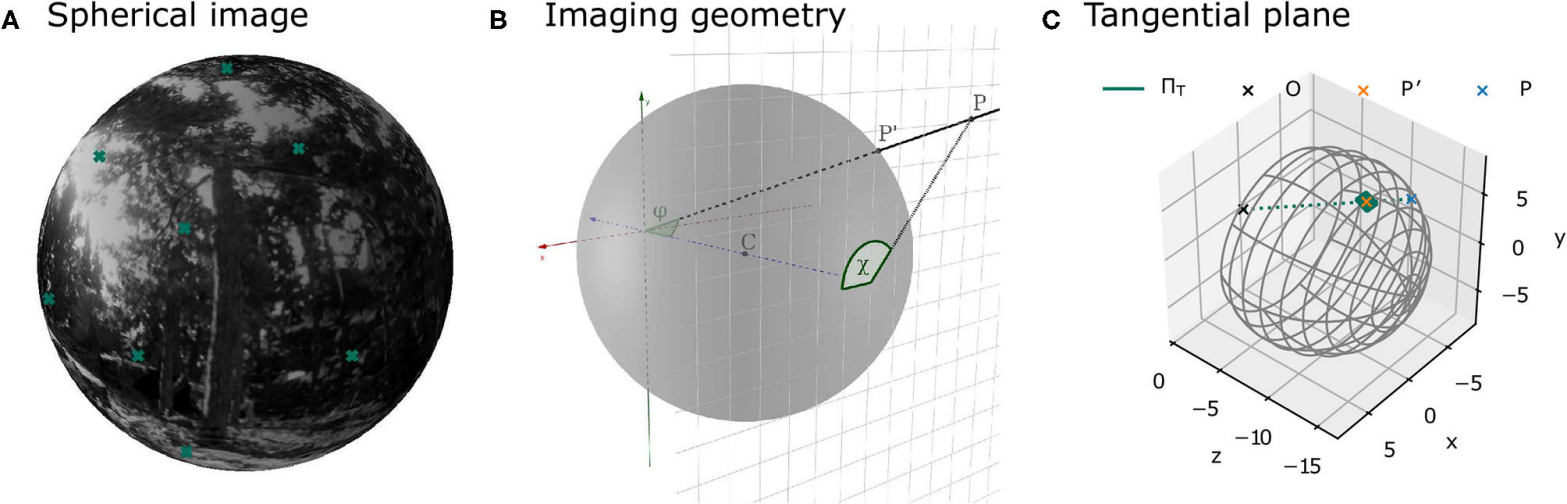
Illustrations of image projections. **(A)** Projection of a sample environment onto a spherical model retina. The crosses indicate the centers of the patches at different positions in the visual field as defined in Figure 4. **(B)** Illustration of the geometry of the imaging process. The image plane at *z* = −2*r* is shown as a grid. Point *p* lies in the image plane. The corresponding point on the sphere is *p*′. The eccentricity φ is the angle between the vector from the origin to the point *p* and the *z*-axis. The polar angle χ is the angle between the point *p* and the *x*-axis on the image plane. **(C)** The local approximation of the sphere by means of a tangent plane is used in image rendering.

**Figure 4:**
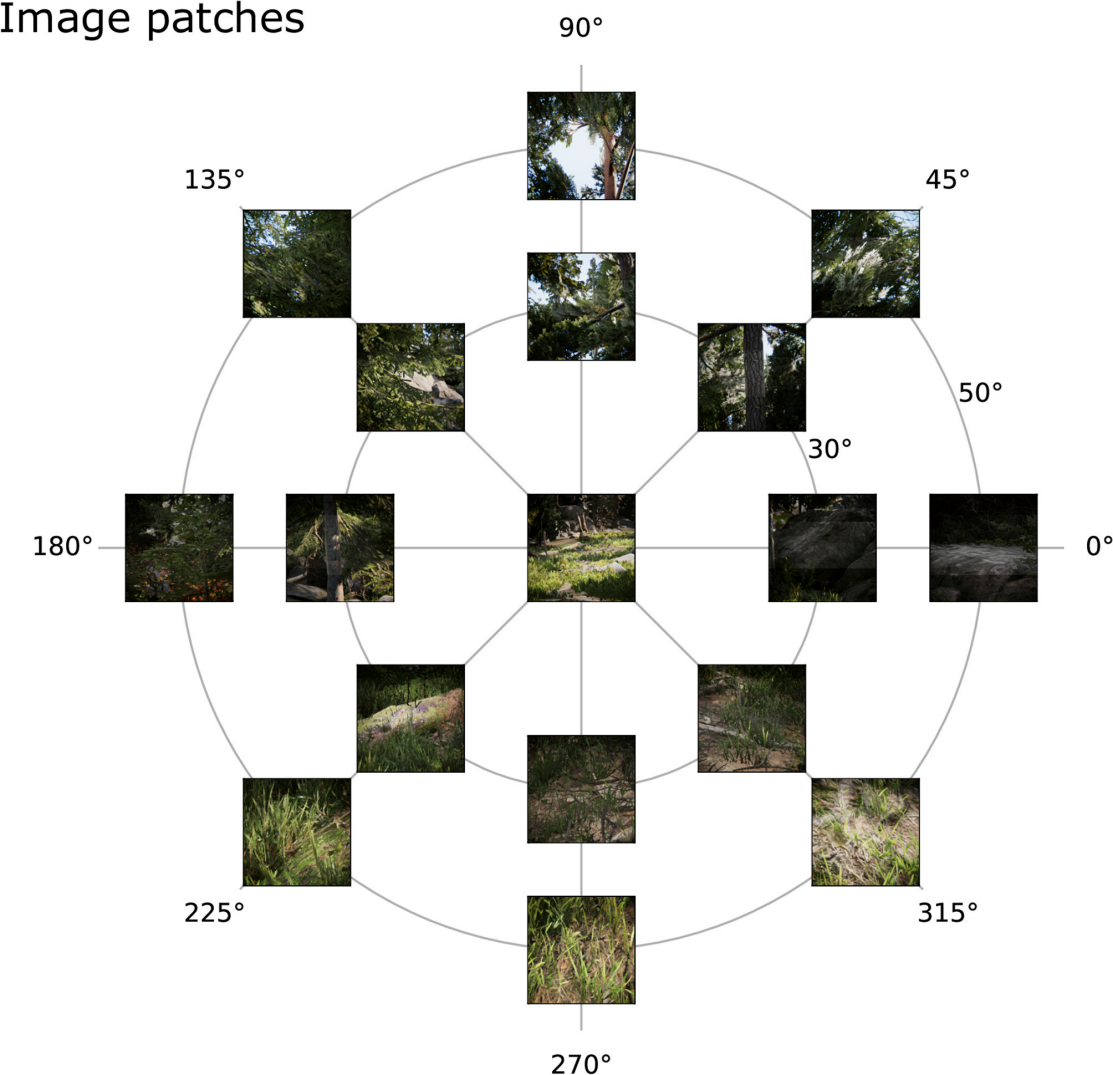
Example image patches. Image patches from the virtual environment across the visual field from the human viewpoint at **(A)** 0°, **(B)** 30°, and **(C)** 50° eccentricity and 8 different polar angles (0–315°) obtained by the simulated imaging process.

The camera was placed at the recorded positions along the trajectory and rotated according to the corresponding orientation of the three different virtual agents. At each of these positions, image patches were sampled from the virtual scene. The parameters of the imaging process were the same as in Pamplona et al. (2013). The field of view covered 120° horizontally and vertically and the focal length was chosen to match the parameters of a thin lens equivalent model of the human or cat eye (see section 2.1.6). Specifically, the environment was projected onto a plane at the back of a model eyeball, with radius *r*, centered at *z* = −*r* in a 3D coordinate system relative to the camera position and rotation. Thus, the environment was projected onto the plane *z* = −2*r* (see Figure 3B). The overall resolution of the entire scene was 5,954 × 5,954 pixels. This resolution was adopted in order to have a resolution similar to van Hateren and van der Schaaf (1998) in the central area of the visual field. Since this resolution is too high to efficiently save and store the full images, image patches of 512 × 512 pixels were sampled at different positions across the visual field (see Figure 4). The coordinates of these positions are specified in terms of eccentricity φ and polar angle χ (see Figure 3B for a definition of the angles). The Cartesian coordinates of a point *P* = [*x, y, z*] on the image plane can be obtained from these angles as follows:

(1) $$\begin{array}{l} \begin{array}{c} x=2r\tan\varphi \cos\chi \\ y=2r\tan\varphi \sin\chi \\ \;z=-2r. \end{array} \end{array}$$

The positions in the visual field, at which image patches were sampled were chosen as the combinations of three eccentricities (φ ∈ {0, 30, 50}) and 8 polar angles, i.e., angles in the *xy*-plane (χ ∈ {0, 45, 90, 135, 180, 225, 270, 315}). Since the coordinate is the same at eccentricity 0 for each polar angle, this results in the 17 combinations in Figure 4, which shows a few exemplary patches. In order to capture the image patch at (φ, χ), the center of the patch was computed according to Equation ((1)) and the projection matrix of the camera was set using a call to OpenGL's frustum($x-\frac{s}{2}$,$x+\frac{s}{2}$, $y-\frac{s}{2}$, $y+\frac{s}{2}$), where *s* is the size of an image patch. In total, 663 × 17 = 11.271 image patches were recorded.

#### 2.1.6. Projection Onto the Retina

The usual imaging model in computer vision applications assumes a planar image, i.e., an image of the world where every world point has been projected onto a plane. While this assumption is valid for camera images, the human eye's surface is not planar. In order to take a step toward a realistic model of the human visual system, the retina can be modeled as a spherical surface. Instead of modeling the lens properties of the optical apparatus and the aberrations introduced by it, an equivalent thin lens model of the human eye with the same parameters as Pamplona et al. (2013) was used. This model assumes that the eye is focused at infinity, resulting in a focal length of *f* = 2*r*, where *r* is the radius of the eye. Each point in the 3D world is then projected onto a sphere, whose center is at *c* = [0, 0, −*r*]. To compare image statistics from the viewpoint of a human and a cat, different parameters of the eye model were chosen for these two viewpoints. For the human model, the eye height was 1.8 m and the focal length was 16.67 mm (Emsley, 1948), while for the cat model the eye height was 0.25 m and the focal length was 12.5 mm (Vakkur and Bishop, 1963).

Figure 3A shows an example image from the virtual environment projected onto a sphere. The coordinates of a point *p*′ = [*x*′, *y*′, *z*′] on the sphere can be obtained from its eccentricity φ and polar angle χ (derivations the Supplementary Material):

(2) $$\begin{array}{l} \begin{array}{c} x^{\prime }=2r\sin\left(\varphi \right)\cos\left(\varphi \right)\cos\left(\chi \right)\\ y^{\prime }=2r\sin\left(\varphi \right)\cos\left(\varphi \right)\sin\left(\chi \right)\\ \;z^{\prime }=-2r\cos^{2}\left(\varphi \right). \end{array} \end{array}$$

Since points on the sphere can either be described using two angles (e.g., in terms of eccentricity and polar angle, see Figure 3B) or using three-dimensional Cartesian coordinates, they are not suitable for analysis with common image processing software, which assumes images with 2D coordinates (i.e., images defined on a plane). As a solution, one can approximate the spherical image locally using a tangent plane (see Figure 3C). For each image patch on the projective plane at the back of the sphere at a specific eccentricity φ and polar angle χ, a corresponding tangent plane is defined by the point $p_{T}^{\prime }$ on the sphere at (φ, χ) and the normal vector $\vec{n}=p_{T}^{\prime }-c$. The point $p_{T}^{\prime }$ then lies both on the sphere and on the tangent plane, while all points in its vicinity are well-approximated with the tangent plane. With increasing image patch sizes, the exactness of the approximation decreases. For this reason and due to computational constraints, an image patch size of 128 pixels on the tangent plane was chosen. This patch size results in a maximal approximation error in 3D space between the sphere and the tangent plane of 0.054 mm at φ = 30° and 0.075 mm at φ = 50°.

The local approximation of the spherical image by a tangent plane has one additional advantage because it allows us to transform the planar image into the approximate spherical image with standard computer vision methods, without first computing the actual spherical image. Two planar images taken from the same camera center are related by a homography, i.e., a 3D projection matrix with 8 degrees of freedom (e.g., Hartley and Zisserman, 2003). A point in homogeneous coordinates on the tangent plane *q*′ = *Ap*′ can be computed from its counterpart on the projective plane *q* = *Bp* with the following equation:

(3) $$\begin{array}{l} \;p^{\prime }\propto p\\ \Rightarrow q^{\prime }\propto A^{-1}Bq\\ \Rightarrow q^{\prime }=Hq, \end{array}$$

where the scaling of *H* is arbitrary. See Supplementary Material for derivations of the matrices *A* and *B*.

Compared to the projection used by Pamplona et al. (2013), this has one advantage in terms of computational efficiency. Pamplona et al. (2013) first compute the spherical image and then reproject it to a plane using the stereographic transform. While this mapping is nonlinear, the approximation introduced here can be modeled with only linear transformations. An image patch on the projective plane can be transformed into its corresponding tangential image patch by warping the image according to the homography matrix *H*. The warping was implemented using the scikit-image package for Python (Van der Walt et al., 2014).

### 2.2. Image Statistics

To evaluate differences between simulated image ensembles we computed two feature statistics that have been highly influential in perceptual science and neuroscience: power spectra and edge histograms.Each of these is briefly described in the following subsections. Prior to these analyses, all image patches were converted to grayscale: they were first transformed to XYZ space and only the luminance channel was used. Then, each image patch was divided by its mean luminance. Statistical tests and circular statistics were computed using the pingouin Python package (Vallat, 2018).

#### 2.2.1. Power Spectra

The second-order statistics of the image patches were quantified using their power spectra. The power spectrum of an image *I*_*i*_(*x, y*) is defined as the squared absolute value of its Fourier transform. The images were preprocessed using a procedure based on the one described by van der Schaaf and van Hateren (1996). Boundary effects of the rectangular image grid were attenuated using a two-dimensional radially symmetric Hamming window *w*(*x, y*). Each image was normalized by subtracting and dividing by the weighted average $\mu_{i}=\frac{\underset{\left(x,y\right)}{\sum }I_{i}\left(x,y\right)w\left(x,y\right)}{\underset{\left(x,y\right)}{\sum }w\left(x,y\right)}$ before applying the window. In summary, the average power spectrum over a set of *N* images was computed as

(4) $$\begin{array}{l} \Gamma \left(u,v\right)=\frac{1}{N}\overset{N}{\underset{i=1}{\sum }}|\underset{\left(x,y\right)}{\sum }\frac{I_{i}\left(x,y\right)-\mu_{i}}{\mu_{i}}w\left(x,y\right)e^{2\pi i\left(ux+vy\right)}{|}^{2}, \end{array}$$

where (*u, v*) are coordinates in the frequency space. They can more intuitively be understood in polar coordinates $\left(f,\theta \right)=\left[\sqrt{u^{2}+v^{2}},atan2\left(v,u\right)\right]$, where *f* is radial spatial frequency and θ the orientation. The size of the image patches on which we computed the power spectra were 128 × 128 pixels and the Hamming window was of the same size.

#### 2.2.2. Parametric Power Spectra Fits

For a more concise representation of the power spectra, they were fit with a parametric function that captures some of the relevant aspects of their shape, such as the characteristic power law shape and an orientation bias. Following Pamplona et al. (2013), an oriented elliptical power law function was used:

(5) $$\begin{array}{l} \Gamma \left(u_{r},v_{r}\right)=\frac{A}{{\left(u_{r}^{2}+v_{r}^{2}b\right)}^{\beta }}, \end{array}$$

where (*u*_*r*_, *v*_*r*_) = [*u* cos(θ) + *v* sin(θ), − *u* sin(θ) + *v* cos(θ)] are the rotated coordinates. This function describes the power spectra as an ellipse, which falls off toward the higher frequencies with a power law scaling β. Setting β = 1 corresponds to the typical $\frac{1}{f^{\alpha }}$ power law with α = 2. The ellipse is rotated by the angle θ, which characterizes the direction of an orientation bias, while the elongation of the ellipse *b* indicates the strength of the orientation bias. In contrast to Pamplona et al. (2013), a hyperbolic component was not needed to adequately characterize the power spectra.

The best-fitting parameters were obtained with least-squares optimization using standard optimization routines from the scipy package. The parameters were optimized within the bounds 0 < θ < π, 1 × 10^−3^ < *A* < 1, 1 × 10^−3^ < *b* < 1, and 0.5 < β < 2.

#### 2.2.3. Edge Orientation Histograms

As an additional method for estimating local orientation content, edge orientation histograms were computed using the method described by Girshick et al. (2011). Specifically, rotationally invariant derivative filters (Farid and Simoncelli, 2004) were used to compute an orientation tensor (see e.g., Granlund and Knutsson, 2013) and, subsequently, its eigenvalue decomposition. Then, pixels whose energy (sum of the eigenvalues) was above the 68th percentile in an image and whose orientedness (eigenvalue difference divided by its sum) was higher than 0.8 were selected as edge pixels. The distributions of the orientations of these edge pixels were aggregated across all image patches in a visual field segment.

To quantify the average orientation in a visual field region, von Mises distributions were fit to the distributions of angles with maximum likelihood estimation. The von Mises distribution can be characterized by two parameters, its mode μ and a measure of concentration κ. Since the angles of oriented edges range from 0 to 180°, the angles were multiplied by 2 prior to fitting. The obtained parameters μ and κ were then divided by 2 in order to be interpreted in the original space of oriented edges. The parameters μ and κ indicate the direction and strength of the orientation bias. In some cases, the distributions were not well-characterized by a single von Mises distribution. Then, a mixture of two von Mises distributions was fit to each of the histograms using expectation maximization (Banerjee et al., 2005).

## 3. Results

### 3.1. Statistics in the Central Area of the Visual Field

**Figure 5:**
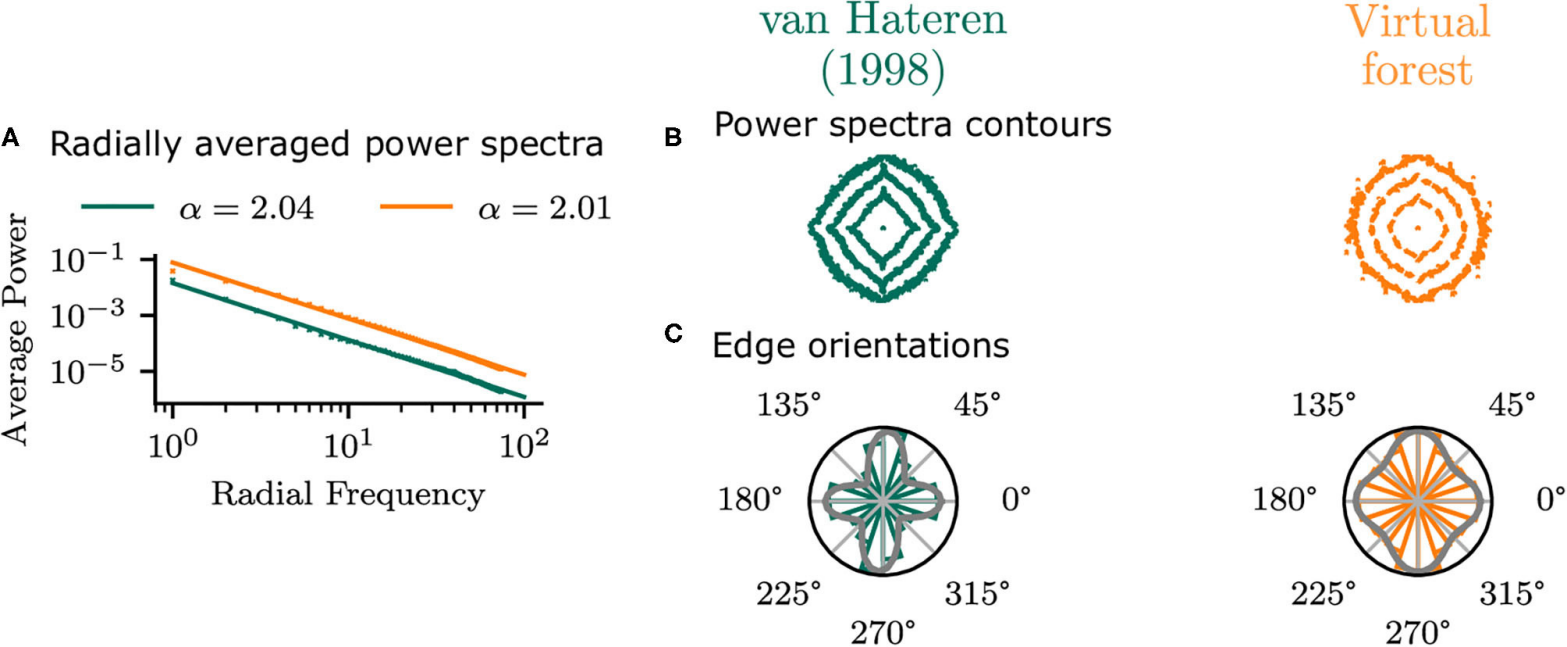
Statistics of planar virtual images in the central area of the visual field compared with those from the van Hateren and van der Schaaf (1998) dataset. **(A)** Shows the radial average power spectra. **(B)** Shows the contours of the power spectra, indicating the 50, 70, and 90% percentiles of the overall log power. Edge orientation histograms are shown in **(C)**, along with the best-fitting mixture of two von Mises distributions.

To establish that the virtual forest scenes do not show idiosyncratic differences to natural images but exhibit similar statistics to real images, the planar virtual images in the central area (φ = 0°, χ = 0°) of the visual field were compared with the van Hateren and van der Schaaf (1998) database. The radially averaged power spectra of the two datasets (Figure 5) have very similar power law exponents, which confirms that the virtual scenes in the current study have similar second-order average statistics to real images.

There are, however, differences in orientation statistics between the virtual forest and the natural scenes. While the natural scenes have a strong bias in the cardinal (horizontal and vertical) directions, the virtual scenes show a weaker cardinal bias. This can be seen in the contour plot of the power spectra, as well as in the edge orientation histograms (Figure 5). The means of a mixture of two von Mises distributions fit to the histograms were −1 and 90° for the virtual images and 0 and 86° for the van Hateren database, indicating a bias toward the cardinal orientations in both cases. The standard deviations of the mixture components are higher for the virtual scenes (19 and 17°) compared to the natural scenes (22 and 23°), which indicates that the edge orientations in the virtual images are more uniformly distributed compared to the real images.

### 3.2. Power Spectra and Orientation Histograms Reveal a Radial Bias

**Figure 6:**
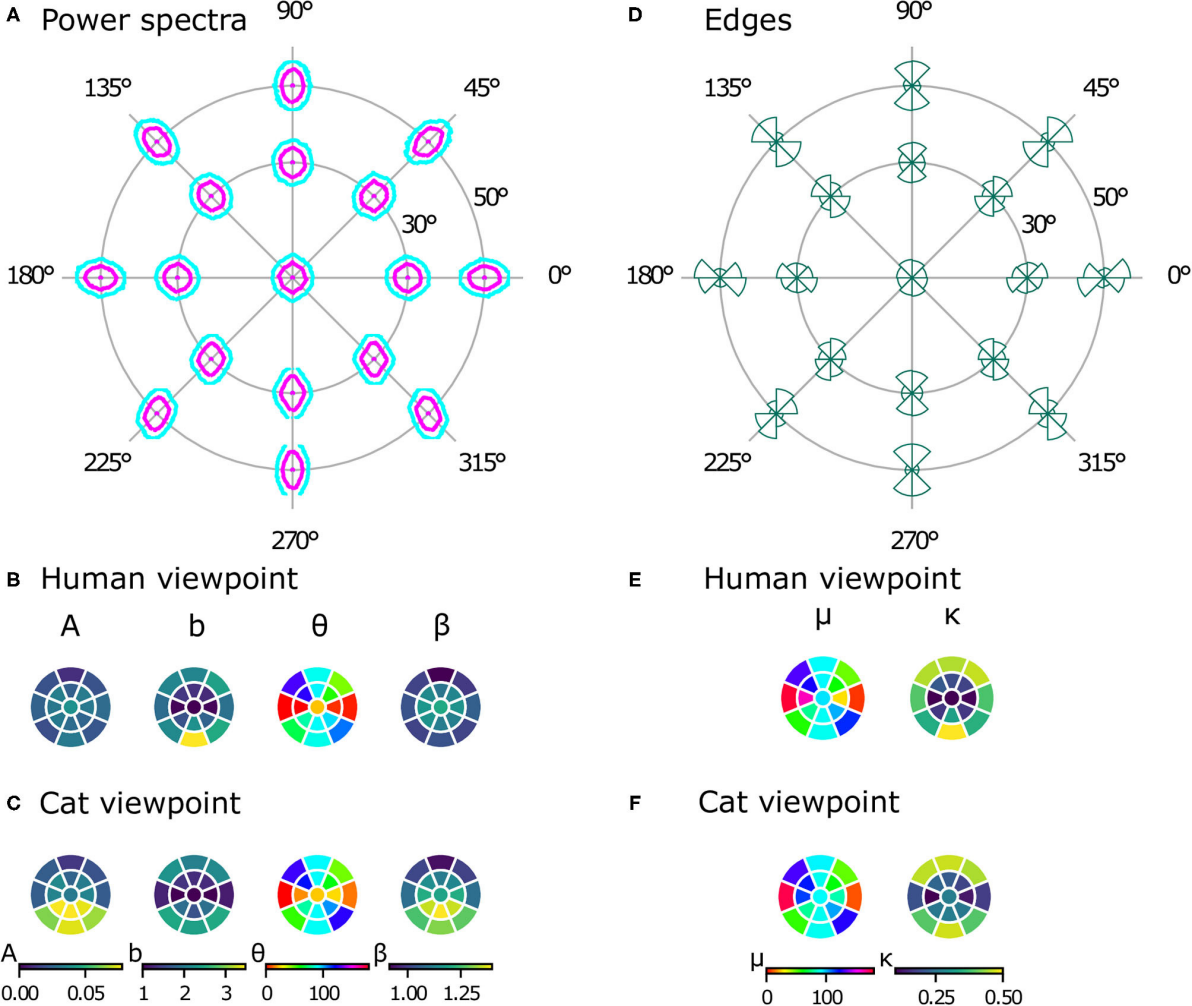
Powers spectra and edge histograms across the visual field. Contour plots indicating 50 and 80% log power are shown at 17 positions across the visual field in **(A)**. Parameters of least-squares fits of ellipses for the power spectra are shown in **(B)** for the human model and in **(C)** for the cat model. The color in a segment of the circle indicates the best-fitting parameter value at that position in the visual field. Edge orientation histograms with maximum likelihood von Mises fits are shown in **(D)**. The mean μ and precision κ of the von Mises fits are shown in **(E)** for the human viewpoint and in **(F)** for the cat viewpoint. The color in a segment of the circle indicates the best-fitting parameter value at that position in the visual field.

While natural image databases cover only the central visual field, our virtual images allow us to analyze image statistics across the whole visual field, revealing differences at different positions in several aspects. Just visually inspecting the power spectra (see Figure 6A) reveals two phenomena. First, there is an obvious radial orientation bias: the best-fitting orientation parameters θ of the power spectra contours (Figure 6B) are highly correlated with the polar angle of the position in the visual field χ (ρ_*c*_ = 0.899, *p* = 0.004). Similarly, the means of the edge distributions μ (Figure 6D) match the respective position in the visual field (ρ_*c*_ = 0.860, *p* = 0.003). Second, the radial bias becomes stronger with increasing eccentricity. This can be seen in the elongation parameter *b* of the power spectra contours (Figure 6B), which becomes larger at higher eccentricities, as does the concentration κ of the edge distributions (Figure 6E). For both parameters, the values are significantly different between 30 and 50° of eccentricity [*t*_(7)_ = −9.18, *p* < 0.001, *d* = 2.70, *CI* = [−1.73, −1.02] for the elongation of the power spectra and *t*_(7)_ = −16.57, *p* < 0.001, *d* = 3.21, *CI* = [−0.65, −0.49] for the concentration of the edge histograms].

### 3.3. Effect of the Spherical Projection

**Figure 7:**
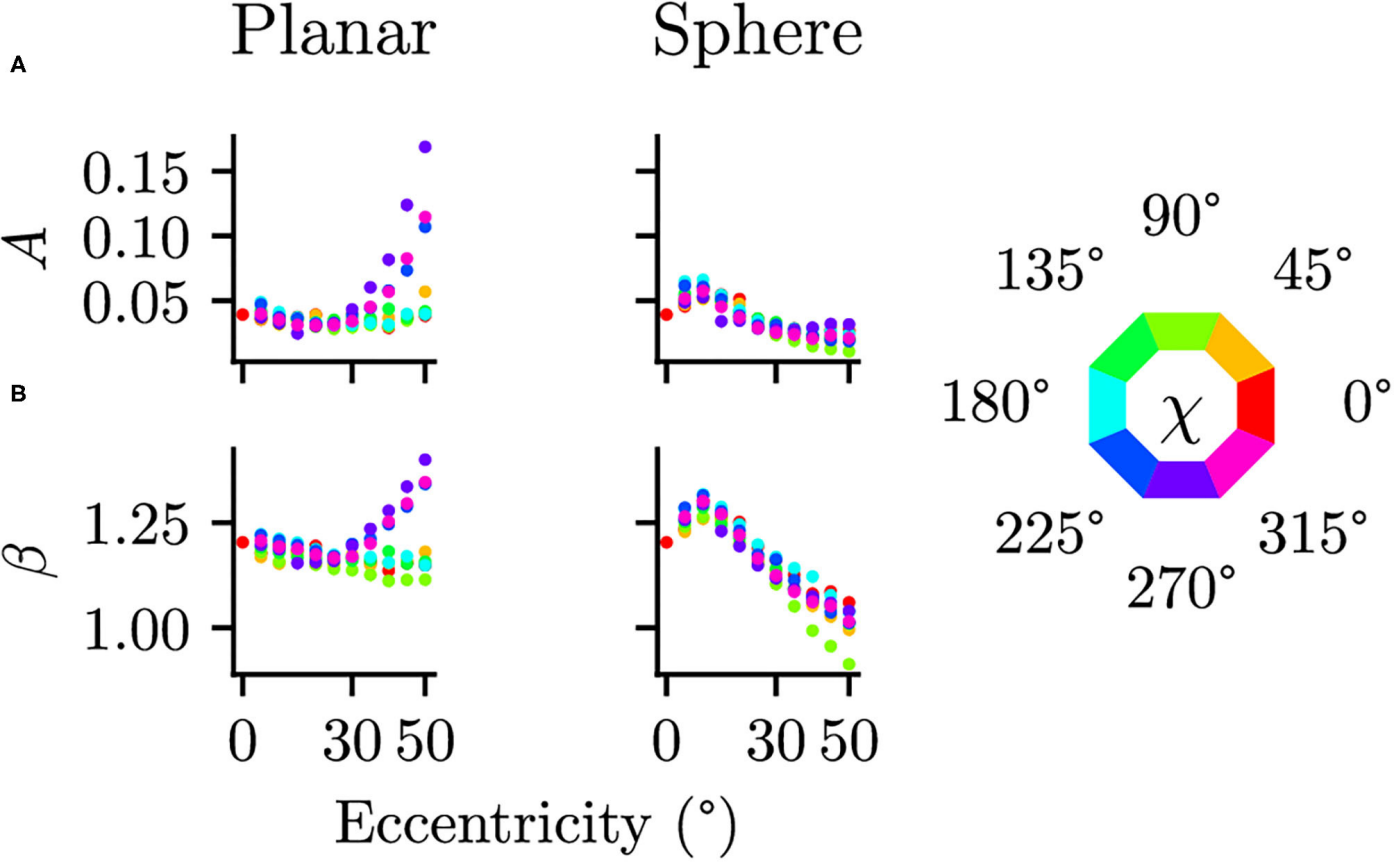
Comparison of planar and spherical projection on power spectra parameters **(A)** The parameter *A* describing the magnitude of power spectra and **(B)** the parameter β describing the exponent in the power law behavior are compared between regular planar projections as in photographic images and spherical projections as on a spherical retina. The color indicates polar angle of the visual field position (see legend on the right).

Differences between the planar and spherical images were quantified by comparing the parameters of the power spectra fits at the 17 positions in the visual field. There were no significant differences in the orientation θ [*t*_(16)_ = 1.073, *p* = 0.300, *CI* = [−4.91, 14.98]] or the shape of the ellipse *b* [*t*_(16)_ = −0.272, *p* = 0.789, *CI* = [−0.08, 0.06]], suggesting that the projection does not change the strength of the radial bias. There are, however, significant differences between planar and spherical images for the power law exponent β [*t*_(16)_ = 3.218, *p* = 0.005, *CI* = [0.03, 0.13]] and the overall power *A* [*t*_(16)_ = 2.967, *p* = 0.009, *CI* = [0.01, 0.05]]. To further analyze these deviations, the parameters *A* and β of the power spectra fits at additional, more fine-grained eccentricities are shown in Figure 7. In the planar images, one can clearly see a different pattern in the β and *A* values between the upper and lower half of the visual field. This difference between the lower and upper visual field is mitigated in the spherical images.

### 3.4. Differences Between Cat and Human Viewpoint

Like the images from the human viewpoint, the images from the cat viewpoint show a radial bias. The main orientation of the ellipse fit to the power spectra (Figure 6C) is highly correlated with the position in the visual field (ρ_*c*_ = 0.732, *p* = 0.007). The picture is less clear for the edge distributions, however (Figure 6F), where the correlation between χ and μ is not as strong but still significant (ρ_*c*_ = 0.602, *p* = 0.045). The strength of the radial bias (quantified by the parameters *b* of the power spectra and κ of the edge histograms) is not significantly different between the cat and human models [*t*_(16)_ = 1.71, *p* = 0.11, *CI* = [−0.04, 0.35] for b, *t*_(16)_ = 0.08, *p* = 0.93, *CI* = [−0.05, 0.05] for κ].

There are, however, differences between image statistics from the human and cat viewpoint. While the power spectra from the human viewpoint are rather symmetric with respect to the horizontal axis, the power spectra from the cat's perspective show a distinct difference between the top and bottom half of the visual field (see Figure 6C). First, as quantified by the parameter *A*, the lower part of the visual field has more overall power. As a result, the values for *A* from the cat model are significantly different from those of the human model [paired *t*-test, *t*_(16)_ = 2.97, *p* = 0.009, *d* = 0.96, *CI* = [0.01, 0.03]]. Second, the parameter β shows that the power spectrum falls off faster with spatial frequency in the lower part of the visual field compared to the upper part. These parameter values are also significantly different from those of the human model [paired *t*-test, *t*_(16)_ = 3.63, *p* = 0.002, *d* = 0.92, *CI* = [0.04, 0.16]].

### 3.5. Differences Between Visual Strategies

**Figure 8:**
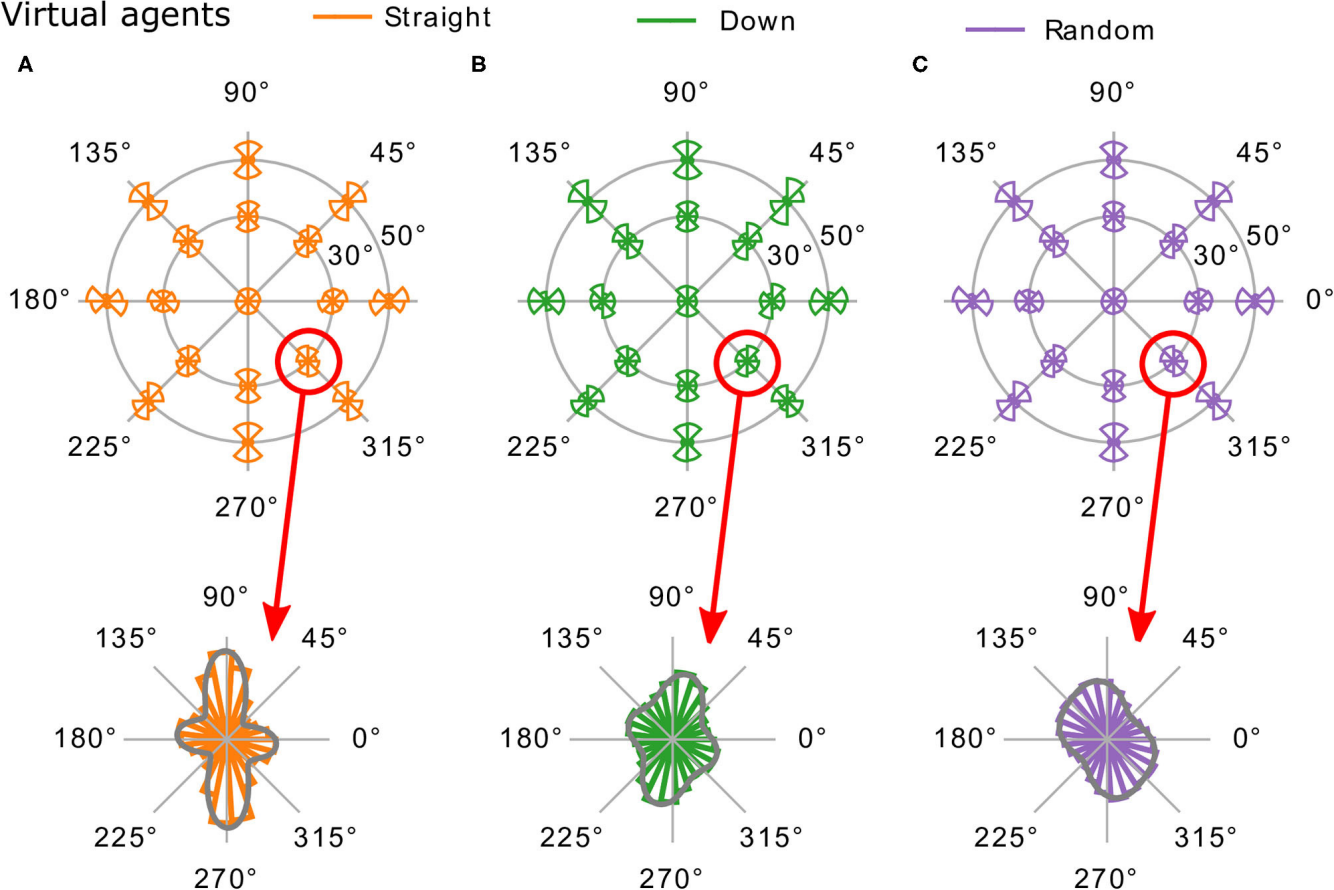
Comparison of edge orientations. Edge orientation distributions across the visual field for the three active vision strategies **(A)** “straight,” **(B)** “down,” and **(C)** “random” are shown in the top row. In the bottom row, the orientation distributions at φ = 30, χ = 315 are shown in more detail (with a mixture of two von Mises distributions fit to the data). Note the differences in orientation and magnitude between the three active gaze behaviors.

In this section, we investigate whether there are differences in the image statistics depending on the visual strategy. The distributions of local edge orientations at 17 visual field positions for all three virtual agents are shown in Figure 8. We compared the means of the orientation distributions between the three strategies using Watson-Williams tests (Berens et al., 2009) and found significant differences at every visual field position (*p* < 0.01). However, visual inspection of the histograms shows that the orientation distributions are not well-described with a single mean direction. We additionally performed pairwise comparisons between the three strategies at each visual field position using non-parametric two-sample Kuiper tests (Kuiper, 1960). Again, all comparisons were significant (*p* < 0.01).

To inspect the differences more closely, we fit mixtures of two von Mises distributions to the orientation histograms and picked out one position in the visual field where the differences are particularly strong: the lower right (φ = 30, ξ = 315). In the images from the *straight* agent, there is a stronger bias toward vertical and horizontal orientations. The means of the mixture components are 91 and 174° (standard deviations 16 and 21°), indicating most notably a cardinal bias. The images from the *random* agent, on the other hand, show a radial bias (similar to the human agent analyzed in section 3.2) and a relatively uniform distribution. Its two mixture components both lie around the radial direction of 135° (98 and 166°) and have relatively high standard deviations (23 and 24°). For the *down* agent, the local orientation distribution is quite different compared to the human and the other two virtual agents. Since the agent looked toward the ground, the visual input is dominated by the ground texture, which consisted of elements oriented toward a vanishing point (quantified by one mixture component's mean at 158°, standard deviation of 23°), and grass elements roughly perpendicular to that (mixture component mean at 76°, standard deviation of 20°).

Note that these results are due to an interaction between visual strategy and the environment: an agent looking straight ahead in a forest environment has a lot of trees in their central visual field (resulting in a cardinal orientation bias), while an agent with a different viewing strategy can have quite different local orientation content.

## 4. Discussion

If the visual system is shaped by the statistics of its input at developmental and evolutionary timescales it is crucial to characterize the statistics of its sensory input signals (Attneave, 1954; Barlow, 1961; Simoncelli and Olshausen, 2001). Both neuronal representations as well as psychophysical performance have been related jointly to measured image statistics (Ganguli and Simoncelli, 2014; Wei and Stocker, 2017). To measure these statistics, several images ensembles from natural environments have been collected (e.g., van Hateren and van der Schaaf, 1998; Tkačik et al., 2011; Adams et al., 2016). Furthermore, some studies utilized video sequences recorded from the head perspective of animals or humans moving through natural environments (e.g., Betsch et al., 2004; Raudies et al., 2012; Orhan et al., 2020) or simulated the physical properties of the visual system (e.g., Nevala and Baden, 2019; Tedore and Nilsson, 2019) when capturing such image collections. With the present study, we move closer to a quantitative understanding of the relative contributions of different factors to the statistics of the natural input to the visual system. Using virtual human and cat avatars moving through a simulated wooded environment, images were sampled at different positions in the visual field. This allowed quantifying the second-order statistical luminance dependencies and the image edge content depending on the position in the visual field, the human and cat viewpoints and image projections, and different active vision sampling strategies.

The statistics of the images in the central area of the visual field, as quantified by power spectra and edge histograms, are comparable with those of photographs in classic natural image databases. This is in accordance with previous research (Rothkopf and Ballard, 2009), which established that the second-order statistics of virtual images resemble those of natural images. The power spectrum shows the typical dependence on spatial frequencies with an exponent of approximately 2. A cardinal bias is present in both second- and higher-order statistics, although the distribution of orientations is more uniform in the virtual forest scenes than in general natural images. This result is most likely due to the type of scene, i.e., forest vs. general landscapes, instead of an artifact of the virtual image generation process, since it agrees with what is known about the spectral contour shapes of different scene categories in static images (Torralba and Oliva, 2003) and during natural behavior (Schumann et al., 2008).

Considering image statics when moving out from central region of the visual field into the periphery provides a much more intricate picture. The cardinal bias changes with increasing eccentricity, but differently depending on the simulated visual behavior. Compared to an actor with a random viewing direction, there is a stronger vertical bias when looking straight ahead and a bias toward the bottom of the visual field when looking toward the ground. This shows that not only the viewpoint but also the active behavior of an actor interacting with the environment shape the statistics of the input to the visual system. Additionally, the cardinal bias is accompanied by a radial bias, which again differs quantitatively between simulated visual behaviors. This radial bias in second-order statistics (Bruce and Tsotsos, 2006; Pamplona et al., 2013) as well as edge orientations (Rothkopf et al., 2009) was present regardless of whether a planar projection or a projection onto a spherical retina was used. Note that this radial bias is not a feature of the virtual environment, but rather due to the perspective projection, since it also occurs projections of randomly oriented edge elements (Pamplona et al., 2013) and real images (Bruce and Tsotsos, 2006).

Consistent with previous research (Pamplona et al., 2013), modeling the eye's geometric properties by projecting the input onto a sphere changes the second-order statistics compared to planar images. Specifically, the amplitude of power spectra increased much less in spherically projected images compared to planar images with increasing eccentricity from the fovea. A second difference between the two projections is the exponent of the power spectrum dependence on spatial frequencies. The commonly found exponent of 2 when rotationally averaging power spectra close to the fovea increases with eccentricity for planar images but decreases for spherically projected images. Thus, the geometry of the retina influences image statistics through the projection onto its surface and therefore needs to be taken into account when relating image statistics to neuronal representations and psychophysical performance.

A comparison using images from a human viewpoint with a cat viewpoint shows comparable edge statistics and power spectra for the upper half of the visual field. By contrast, differences between the upper and lower visual field are more pronounced in the input to the cat's visual system. Particularly, the overall power spectrum amplitude is higher in the lower area of the cat's visual field. This result is qualitatively consistent with results on actual videos taken from a cat's viewpoint (Betsch et al., 2004). The reason is most likely the higher density of edge elements in images taken from the viewing height of the cat avatar because of the smaller distance to objects on the ground plane.

Taken together, comparing image statistics obtained by simulating different visual behaviors in human and cat avatars in a naturalistic wooded environment revealed quantitative differences in power spectra and edge content across the visual field. This is not necessarily obvious, as the different influences could potentially average out over large image ensembles. This result implies that models investigating the relationship between image statistics, neuronal representations, and psychophysical performance (e.g., Ganguli and Simoncelli, 2014; Wei and Stocker, 2017) in humans and animals should increase validity by using representative image ensembles. Accordingly, such image ensembles may show substantial differences in the quantitative results explaining properties of retinal ganglion cells in terms of second order image statistics such as the extent of center-surround antagonism or elongation of profile (Atick and Redlich, 1992; McIntosh et al., 2016; Ocko et al., 2018). Similarly, properties of V1 simple cells in terms of higher order statistical dependencies in natural images such as preferred orientation or spatial frequency preferences may be affected (e.g., Olshausen and Field, 1996; Güçlü and van Gerven, 2015; Chalk et al., 2018; Cadena et al., 2019). By contrast, most current studies investigating the relation between natural image statics and neuronal representations through statistical learning utilize generic image databases (e.g., Yamins et al., 2014; McIntosh et al., 2016; Cadena et al., 2019; Lindsey et al., 2019). Therefore, future work of computational models of visual representations should take into account the statistics of actors' behaviors in their natural environment and how these interact with the environmental regularities in producing the statistics of the natural input to the visual system.

### 4.1. Limitations

First, only a single type of virtual environment, specifically a forest scene, was used in generating the images. As discussed above, second-order statistics and edge histograms show that the distribution of orientations is more uniform in a forest than in other types of natural environments. Accordingly, further investigations on the influence of properties of the environment on image statistics are required potentially also using simulations of avatars in virtual environments. Furthermore, a lot of humans' visual experience is made in man-made environments and during the interaction with objects or other people. Torralba and Oliva (2003) have shown that the low-level statistics of natural landscapes and man-made environments are very different. Future work should take this into account and investigate how active vision influences the input statistics while interacting with objects or people.

Second, only the geometrical optics were modeled, disregarding other factors such as the blurring and additional aberrations induced by the eye's imaging process. In this study, these were not modeled to enable a comparison with the cat viewpoint. Although measurements of the cat's modulation-transfer function exist (Wässle, 1971; Bonds et al., 1972), there is, to our knowledge, no data on how the cat's MTF changes with eccentricity. Nevertheless, the influences of the geometric optics on the projections of scenes onto the retina does not change by including additional blurring due to aberrations and defocus. The the provided databases will allow investigating these influences in the future by including the position-dependent image transformations.

Third, we simulated the natural input to the visual system only up to the level of the retina. If our goal is to quantitatively relate image statistics to biases in psychophysical experiments, further processing steps, such as the receptor density on the retina, processing in the retinal ganglion cells and LGN as well as in early visual cortical areas need to be taken into account. Quantifying the statistics of the natural input to the visual system at these higher processing stages would enable ideal-observer analysis (Geisler, 1989) of more complex stimuli than are typically used in psychophysical experiments.

Finally, although first steps were made toward incorporating behavior by simulating viewing directions of several virtual agents, the current study is only an initial investigation demonstrating the dependence of input statistics on the active use of the sensory system. It is well-known that vision is an active sensory modality that is driven by eye movements and blinking behavior (Findlay and Gilchrist, 2003). Here, we only considered three hand-crafted visual behaviors. Tomasi et al. (2016) have measured how head movements and gaze directions are coordinated in natural environment, and recent work by Thomas et al. (2020) explores how different surfaces influence gaze behaviors. To more accurately quantify the influence of gaze direction on image statistics, the statistics of gaze behaviors from these findings should be incorporated into the simulated strategies. Furthermore, in our work only the head direction was considered during sampling of the environment. While related work (Schumann et al., 2008) has found little difference in power spectra between gaze- and head-aligned images, there were differences in local image features. Complicating the interpretation of such investigations is the low accuracy of current gaze tracking devices relative to visual feature distributions. Future work should investigate how differences in gaze targets due to movements of the body, the head, and the eyes shape the statistics of the natural input to the visual system across the entire visual field.

## 5. Conclusion

The present study investigated the statistics of low-level image features across the visual field using simulations of different active visual strategies in human and cat avatars in a naturalistic virtual environment. Taken together, the results of the present study show that the natural input to the visual system is not only influenced by the structure of the environment, but also by how the environmental statistics interact with the projective geometry of an agent's visual system and its active visual behavior. Future studies linking neural representations, behavior in psychophysical tasks, and natural image statistics should take this into account.

## Data Availability Statement

Code to reproduce the results from this paper is available at https://github.com/RothkopfLab/imgstats-frontiersin.

## Ethics Statement

Ethical review and approval was not required for the study on human participants in accordance with the local legislation and institutional requirements. Written informed consent for participation was not required for this study in accordance with the national legislation and the institutional requirements.

## Author Contributions

CR and DS conceptualized the research. DS generated the image database, performed the data analysis, and wrote the first draft of the manuscript. CR wrote sections of the manuscript. Both authors contributed to manuscript revision, read, and approved the submitted version.

## Conflict of Interest

The authors declare that the research was conducted in the absence of any commercial or financial relationships that could be construed as a potential conflict of interest.

## Footnotes

^1^http://unity3d.com, version 2018.2.21f1.

^2^https://unity3d.com/book-of-the-dead, version 1.1.
