# Supplementary material for: Looking for Image Statistics: Active Vision With Avatars in a Naturalistic Virtual Environment
Source: Front Psychol. 2021 Feb 22;12:641471. doi: 10.3389/fpsyg.2021.641471 (PMC7937646; doi:10.3389/fpsyg.2021.641471)
Supplement: Supplementary file 1 [file Data_Sheet_1.PDF]

Supplementary Material

1 DERIVATIONS

1.1 Spherical coordinates

For points $P = [x, y, z]$ on the projective plane, we have

$$z = -2r.$$

From $\tan\varphi = \frac{w}{zr}$ and $\cos\chi = \frac{x}{w}$ we obtain

$$x = 2r\tan\varphi\cos\chi$$

And, finally, from $\tan\chi = \frac{y}{x}$, we have

$$y = x\tan\chi = 2r\tan\varphi\sin\chi$$

For points $P' = [x', y', z']$ on the sphere, the equations are a little more complicated. First, we obtain the z coordinate via two identities that include $\cos\varphi$. We define a as the distance from the origin to the point on the sphere. Then, from $\cos\varphi = \frac{a}{-2r}$ and $\cos\varphi = \frac{z}{a}$, we get

$$z = -2r\cos^2\varphi.$$

The x' and y' coordinates can be obtained from the z' coordinate as for the planar case:

$$x' = z'\tan\varphi\cos^2\varphi\cos\chi$$
$$y' = x'\tan\chi$$

1.2 Homography

Assume that $q = (q_1, q_2, 1)^T$ is a point in homogeneous coordinates on the projective plane at the back of the model eye. Its 3D coordinates are given by $p = Bq$. The 3×3 matrix B consists of two basis vectors and a point that define the plane. It relates 3D coordinates to points in the plane's coordinate system. For an image patch on the projective plane centered at a given point b_0 (obtained from φ and χ via Equation (**??**)), the matrix is given by

$$B = \begin{pmatrix} 1 & 0 & \vdots \\ 0 & 1 & p_0 \\ 0 & 0 & \vdots \end{pmatrix}. \tag{S1}$$

A point p' on a tangent plane to the sphere can be written in the same way as a matrix-vector product of a matrix A and its homogeneous coordinates $q' = (q_1', q_2', 1)^T$: $p' = Bq'$. Here, finding the basis vectors is a bit more difficult. As a point in the plane, we use the point p_T' at which the plane is tangential to the sphere (i.e. a point on the sphere obtained from φ and χ via Equation (**??**)). From the definition of our

tangential plane in terms of p'_T and a normal vector $\vec{n} = p'_T - c$, we need to obtain vectors inside the plane. To this end, we find the points u_1 and u_2, where a line from the origin to $b_0 + (1,0,0)^T$ or $b_0 + (0,1,0)^T$, respectively, cuts the tangent plane. This yields the basis vectors v_1 and v_2 of the tangent plane, resulting in

$$A = \begin{pmatrix} \vdots & \vdots & \vdots \\ v_1 & v_2 & p'_T \\ \vdots & \vdots & \vdots \end{pmatrix}. \tag{S2}$$

Since the points p and p' lie on the same ray from the origin, they are only different by a constant factor, i.e. $p' \propto p$. By inserting the definitions of p and p' in terms of their respective projection matrices, we obtain

$$q' \propto A^{-1}Bq \tag{S3}$$

$$q' = Hq, \tag{S4}$$

with $H = \alpha A^{-1}B$ with an arbitrary scaling constant α.
